# Severe sepsis in the United Sates: a 5-year analysis

**DOI:** 10.1186/cc10659

**Published:** 2012-03-20

**Authors:** J Knittel, S Quraishi

**Affiliations:** 1 Massachusetts General Hospital, Boston, MA, USA

## Introduction

We describe patient-level healthcare data related to severe sepsis over a 5-year period (2004 to 2008) in the United States.

## Methods

We queried the largest all-payer inpatient care database in the United States to identify cases of hospital admissions between 2004 and 2008 with a primary diagnosis of severe sepsis (ICD 9: 995.92). This retrospective analysis was performed with data from the Healthcare Cost and Utilization Project National Inpatient Sample (NIS) repository. Data related to length of stay, in-hospital mortality, and hospital charge was extracted. The 2004 and 2008 data for these variables were compared and further analyzed by age and sex in SPSS v.19 (IBM Corporation, Amonk, NY, USA). Results are reported with ± standard error where applicable, and *P *< 0.05 represented statistical significance.

## Results

Our query of the NIS data revealed a similar number of hospital admissions with a primary diagnosis of severe sepsis in 2004 versus 2008. Sex (male vs. female) and age group composition (18 to 44 vs. 45 to 64 vs. 65 to 85 vs. 85+) within these cohorts were similar. No significant change in overall length of stay or in-hospital mortality rate was appreciated. However, a significant increase in overall cost was appreciated ($67,670 ± 5,742 vs. $100,973 ± 10,525; *P *= 0.006), which outpaced healthcare-specific and general inflation during this period. Sex did not influence length of stay or in-hospital mortality rate. Cost of care was higher for males versus females (2004: $78,361 ± 8,982 vs. $57,040 ± 5,959; *P *= 0.048 and 2008: $111,298 ± 13,835 vs. $90,730 ± 11,380; *P *< 0.001). Age had a significant influence on in-hospital mortality in 2004 and in 2008, with the highest percentage of in-hospital deaths in the 85+ category. Age also had a significant influence on cost/day. Whereas in 2004 patients in the 85+ category represented the age subset with the lowest cost/day, in 2008 this age group witnessed a threefold increase in daily costs (*P *< 0.001) and represented the highest cost/day subset.

## Conclusion

Our data suggest that despite significant increases in healthcare costs attributable to severe sepsis, survival and length of stay has not improved significantly between 2004 and 2008. Dramatic increases in cost are particularly notable in males versus females and in patients who are 85 years old and over. Policies to control healthcare costs in the United States should focus on the root causes that lead to such significant increases in cost without appreciable societal returns on investment.

